# RETRACTION: Post‐Inflammatory Hyperpigmentation: Molecular Mechanisms and Advances in Dermocosmetic Management

**DOI:** 10.1111/jocd.71159

**Published:** 2026-08-25

## Abstract

**RETRACTION**: LiR. , LiB. , ZhangQ. , ZhaoC. , YeC. , ChenY. , HuguetC. , XiongW. , BeguinV. , KerobD. , and ZhengY. , “Post‐Inflammatory Hyperpigmentation: Molecular Mechanisms and Advances in Dermocosmetic Management,” Journal of Cosmetic Dermatology 25, no. 6 (2026): e70738, 10.1111/jocd.70738.

The above article, published online on 28 May 2026 in Wiley Online Library (wileyonlinelibrary.com), has been retracted by agreement between the authors; the journal Editor‐in‐Chief, Michael H. Gold; and Wiley Periodicals LLC. The retraction has been agreed upon following a request from the authors. The authors acknowledged that not all authors had reviewed and approved the final version of the manuscript prior to submission. Furthermore, the article contains incorrect information, including the misclassification of a UV‐induced pigmentation model as post‐inflammatory hyperpigmentation (PIH), which resulted in a systematic bias in the pooled effect size and the GRADE evidence assessment. The authors acknowledge that this error may mislead clinical decision‐making regarding the efficacy of dermocosmetic products for the treatment of PIH. The editors agree with the authors that retraction of the article is appropriate.

**RETRACTION**: R. Li , B. Li , Q. Zhang , C. Zhao , C. Ye , Y. Chen , C. Huguet , W. Xiong , V. Beguin , D. Kerob , and Y. Zheng , “Post‐Inflammatory Hyperpigmentation: Molecular Mechanisms and Advances in Dermocosmetic Management,” Journal of Cosmetic Dermatology 25, no. 6 (2026): e70738, 10.1111/jocd.70738.

The above article, published online on 28 May 2026 in Wiley Online Library (wileyonlinelibrary.com), has been retracted by agreement between the authors; the journal Editor‐in‐Chief, Michael H. Gold; and Wiley Periodicals LLC. The retraction has been agreed upon following a request from the authors. The authors acknowledged that not all authors had reviewed and approved the final version of the manuscript prior to submission. Furthermore, the article contains incorrect information, including the misclassification of a UV‐induced pigmentation model as post‐inflammatory hyperpigmentation (PIH), which resulted in a systematic bias in the pooled effect size and the GRADE evidence assessment. The authors acknowledge that this error may mislead clinical decision‐making regarding the efficacy of dermocosmetic products for the treatment of PIH. The editors agree with the authors that retraction of the article is appropriate.

